# Synthetic lethal genetic interactions between Rad54 and PARP-1 in mouse development and oncogenesis

**DOI:** 10.18632/oncotarget.10479

**Published:** 2016-07-07

**Authors:** Mirella Tanori, Arianna Casciati, Francesco Berardinelli, Simona Leonardi, Emanuela Pasquali, Francesca Antonelli, Barbara Tanno, Paola Giardullo, Alessandro Pannicelli, Gabriele Babini, Ilaria De Stefano, Antonella Sgura, Mariateresa Mancuso, Anna Saran, Simonetta Pazzaglia

**Affiliations:** ^1^ Laboratory of Biomedical Technologies, Agenzia Nazionale per le Nuove Tecnologie, l’Energia e lo Sviluppo Economico Sostenibile (ENEA), CR-Casaccia, Rome, Italy; ^2^ Department of Science, University Roma Tre, Rome, Italy; ^3^ Department of Radiation Physics, Università degli Studi Guglielmo Marconi, Rome, Italy; ^4^ Technical Unit of Energetic Efficiency, ENEA, Rome, Italy; ^5^ Department of Physics, University of Pavia, Pavia, Italy

**Keywords:** cerebellum, expression profiles, medulloblastoma, apoptosis, senescence

## Abstract

Mutations in DNA repair pathways are frequent in human cancers. Hence, gaining insights into the interaction of DNA repair genes is key to development of novel tumor-specific treatment strategies. In this study, we tested the functional relationship in development and oncogenesis between the homologous recombination (HR) factor *Rad54* and *Parp-1*, a nuclear enzyme that plays a multifunctional role in DNA damage signaling and repair. We introduced single or combined *Rad54* and *Parp-1* inactivating germline mutations in *Ptc1* heterozygous mice, a well-characterized model of medulloblastoma, the most common malignant pediatric brain tumor. Our study reveals that combined inactivation of *Rad54* and *Parp-1* causes a marked growth delay culminating in perinatallethality, providing for the first time evidence of synthetic lethal interactions between *Rad54* and *Parp-1 in vivo*. Although the double mutation hampered investigation of *Rad54* and *Parp-1* interactions in cerebellum tumorigenesis, insights were gained by showing accumulation of endogenous DNA damage and increased apoptotic rate in granule cell precursors (GCPs). A network-based approach to detect differential expression of DNA repair genes in the cerebellum revealed perturbation of p53 signaling in *Rad54*^-/-^/*Parp-1*^-/-^/*Ptc1*^+/-^, and MEFs from combined *Rad54/Parp-1* mutants showed p53/p21-dependent typical senescent features. These findings help elucidate the genetic interplay between *Rad54* and *Parp-1* by suggesting that p53/p21-mediated apoptosis and/or senescence may be involved in synthetic lethal interactions occurring during development and inhibition of tumor growth.

## INTRODUCTION

The complex network of DNA repair system in mammals counteracts genome insult and maintains genomic stability. DNA repair failure increases mutations frequency and affects cell-cycle regulation, promoting tumorigenesis; in fact, inactivating mutations in DNA repair pathways are frequent in cancer. On the other hand, DNA repair defects provide new therapeutic opportunities to treat cancer through DNA-damage-inducing radiation and chemotherapies. Identification of synthetic interactions, *i.e.*, negative genetic combination of mutations in two or more genes, leading to severe slow growth or lethality compared to single mutants, has grown in popularity in the DNA damage field, with the finding that poly(ADP-ribose) polymerase (Parp-1) inhibitors show synthetically lethal effects in homologous recombination (HR) defective tumors [1].

PolyADP-ribosylation acts in DNA-damage repair response and maintenance mechanisms of genomic stability. Several DNA repair pathways, including base-excision repair and double strand break (DSB) repair, involve Parp functions. Inhibition of Parp-1 results in an increase in single strand breaks (SSBs) that then collapse replication forks into toxic one-ended DSBs [1,2], which are substrates for HR [3,4]. In addition, through enzyme-dependent chromatin remodeling and enzyme-independent motif recognition, Parp-1 also plays important roles in regulating gene expression [5]. Furthermore, Parp-1 binding motifs may be readily found in promoter elements of DNA repair genes, linking DNA repair and transcription functions of Parp-1 [5]. The critical role of Parp-1 in maintenance of genomic stability is reflected in its frequent upregulation in cancer [6,7], and by hypersensitivity of *Parp-1* null animals toward the mutagenic effect of DNA damaging agents [8].

The *Rad54* gene encodes a dsDNA-dependent ATPase of the Swi2/Snf2 family [9], which interacts directly with Rad51 [10,11], and stimulates its DNA exchange activity [12]. Rad54 promotes chromatin remodeling, Rad51 displacement from double-stranded DNA, binds Holliday junctions and drives their branch migration [13]. Although disruption of other genes involved in HR leads to embryonic lethality, adult *Rad54*^-/-^ mice are viable and fertile, providing a valuable model to study the effects of attenuated HR. Interestingly, several point mutations in conserved regions of the *Rad54* gene family have been found in primary tumors (*e.g.*, breast and colon carcinomas and lymphomas) [14,15], and approximately half of epithelial ovarian cancers have alterations in genes regulating HR repair [16].

Most DNA repair pathways are complex, involving many proteins working in discrete consecutive steps. Therefore, their balanced and coordinated expression is important to avoid erroneous repair that might result from excessive base removal and DNA cleavage. In addition, as DNA damage is a likely factor in promoting central nervous system (CNS) pathophysiology, DNA repair may be an important mechanism for maintenance of normal physiological function. In this study, to investigate the functional relationship between the HR factor *Rad54* and *Parp-1* in development and oncogenesis *in vivo*, we introduced single or combined *Rad54* and *Parp-1* inactivating mutations in a well characterized cancer model, the *Ptc1* heterozygous mice. Mice in which one copy the *Ptc1* gene has been inactivated (*Ptc1*^+/-^) are developmentally nearly normal, but show a marked predisposition to tumor development, including medulloblastoma (MB), a frequent pediatric malignant tumor of the cerebellum [17,18]. Our previous work showed that neonatal irradiation dramatically increases the frequency of MB [19,20]. We also showed that combined loss of *Ptc1* and *Rad54* or of *Ptc1* and *Parp-1* increased radiation induced MB [21,22]. Here, we analyzed the effects of combined loss of *Rad54* and *Parp-1* in the *Ptc1* mouse model of spontaneous and radiation-induced cancer, highlighting novel synthetic lethal interactions during development and tumorigenesis, and dissecting the underlying cellular and molecular mechanisms.

## RESULTS

### Survival and oncogenesis in crosses between *Rad54, Parp-1* and *Ptc1* mutant mice

Defects in DNA damage signaling are frequently associated with neurodegeneration, neurodevelopmental disease and brain tumors. We therefore sought to determine the oncogenic potential of combined genetic disruption of *Rad54* and *Parp-1* in the CNS by knocking out these genes in *Ptc1*^+/-^ mice. To this aim, *Rad54*^+/-^*/Parp-1*^+/-^*/Ptc1*^+/-^ and *Rad54*^+/-^*/Parp-1*^+/-^*/Ptc1*^+/+^ mice were intercrossed, and the progeny genotyped. For simplification, *wt* genotypes are hereafter omitted from the label, unless differently specified. Mutants for *Rad54* (*Rad54*^-/-^/*Parp-1*^+/-^/*Ptc1*^+/-^) or *Parp-1* (*Rad54*^+/-^/*Parp-1*^-/-^/*Ptc1*^+/-^) were viable. Irrespective of *Ptc1* genotype, no mice with compound *Rad54*/*Parp-1* inactivation were found at weaning, showing strong synthetic lethal interaction between *Rad54* and *Parp-1* genes *in vivo*, in agreement with existing *in vitro* data. To look for haploinsufficient interactions, compound mutant mice with varying gene dosage of *Rad54* and *Parp-1* were analyzed for survival and cerebellum tumorigenesis, with or without irradiation. Because only *Ptc1*^+/-^ mice are prone to MB, the effects of *Rad54* and *Parp-1* inactivation was evaluated only in mice with *Ptc1*^+/-^ genotype.

**Figure 1:**
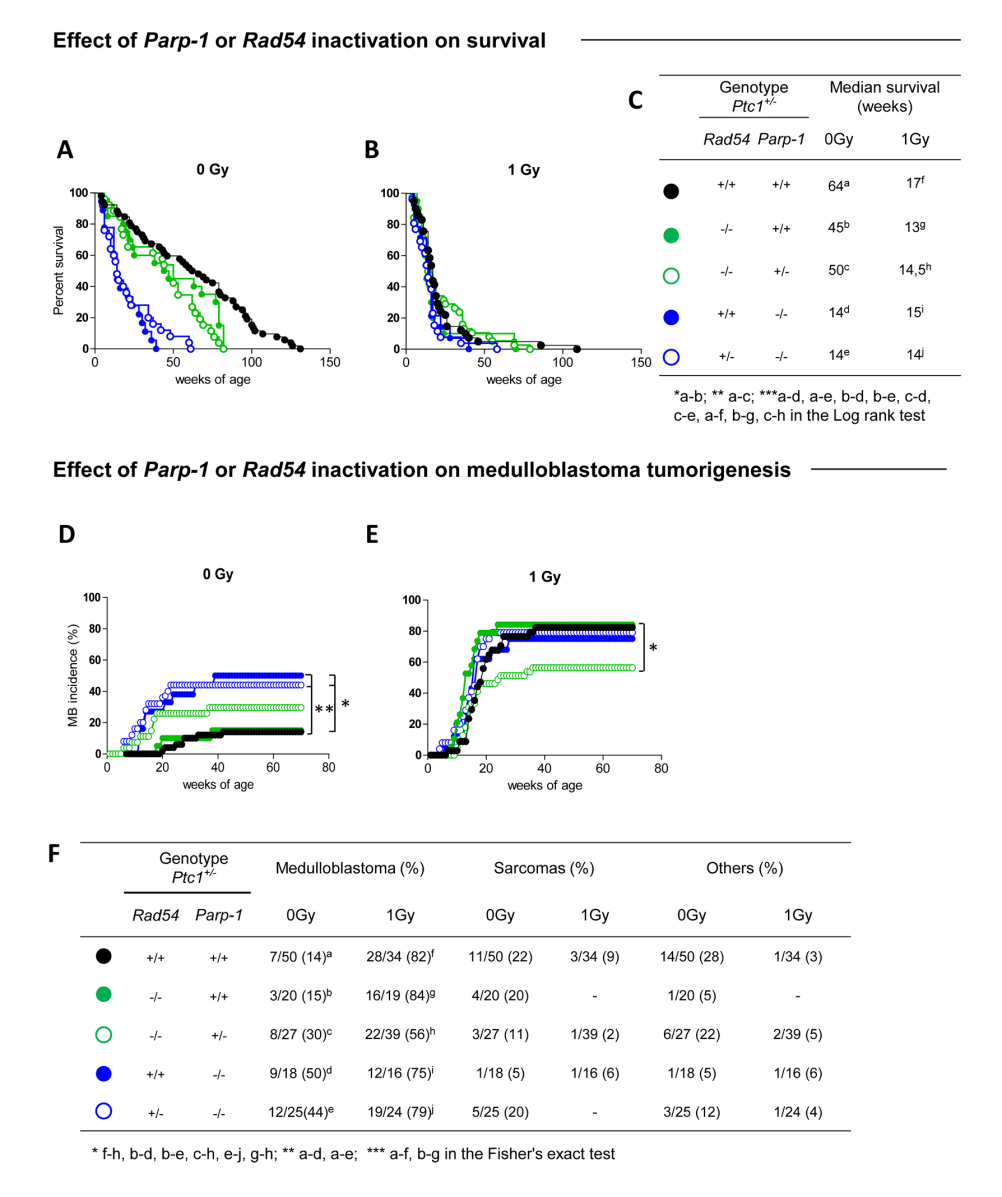
Effect of ***Rad54*** and ***Parp-1*** inactivation on survival and tumor development **(A)** Survival curves of unirradiated *Ptc1*^+/-^ , *Rad54*^-/-^/*Ptc1*^+/-^*, Rad54*^-/-^/*Parp-1*^+/-^/*Ptc1*^+/-^*, Parp-1*^-/-^*/Ptc1*^+/-^ and *Rad54*^+/-^/*Parp-1*^-/-^/*Ptc1*^+/-^ mice, showing significant lifespan shortening after inactivation of *Parp-1*. **(B)** Survival curves of *Ptc1*^+/-^, *Rad54*^-/-^/*Ptc1*^+/-^*, Rad54*^-/-^/*Parp-1*^+/-^/*Ptc1*^+/-^*, Parp-1*^-/-^*/Ptc1*^+/-^ and *Rad54*^+/-^/*Parp-1*^-/-^/*Ptc1*^+/-^ mice irradiated with 1Gy at P1, all showing significant lifespan shortening compared to control mice with exception of *Parp-1* null mice (*Rad54*^+/+ or +/-^/*Parp-1*^-/-^/*Ptc1*^+/-^). **(C)** Median survival of unirradiated and irradiated mice. **(D)** Effect of *Rad54* and *Parp-1* inactivation on spontaneous and **(E)** radiation-induced medulloblastoma tumorigenesis. **(F)** Percent incidence of medulloblastoma, sarcoma and other tumors for each mouse group. *P ≤ 0.05; **P ≤ 0.005; *** *P* ≤ 0.0001.

Lack of *Parp-1*, or *Rad54 per se* affected lifespan of *Ptc1*^+/-^ mice by significantly shortening the median survival time from 64 to 14 (*P* ˂ 0.0001) or 45 weeks (*P* = 0.0206), respectively (Figure 1A and 1C). However, loss of *Parp-1* function had more severe effects on survival compared to *Rad54* loss (i.e., 14 *vs* 45 weeks; *P* = 0.0002) (Figure 1A), suggesting a preferential role for *Parp-1* in resolution of spontaneous DNA damage compared to *Rad54*. Irradiation of *Ptc1*^+/-^ mice or *Ptc1*/*Rad54* compound mutants (*Rad54*^-/-^/*Ptc1*^+/-^) with 1 Gy of x-rays caused a sharp reduction of lifespan relative to controls (17 *vs* 64 weeks *P* ˂ 0.0001; 13 *vs* 45 weeks; *P* = 0.0002; Figure 1A-1C), with median survival reduced approximately 3-fold. Notably, life shortening was not affected by *Rad54* status, in agreement with findings that *Rad54*^-/-^ mice are not hypersensitive to radiation [23]. Moreover, irradiated *Ptc1*/*Parp-1* compound mutants (*Parp-1*^-/-^/*Ptc1*^+/-^) did not show further survival reduction compared to controls (15 vs 14 weeks; *P* = 0.862; Figure 1A-1C). The lack of modifying effects of *Parp-1* on *Ptc1*-associated radiosensitivity is consistent with previous reports from our laboratory [22].

Coherent with survival reduction, genetic disruption of *Parp-1* significantly increased spontaneous MB incidence in *Ptc1*^+/-^ mice (50% *vs* 14% in *Parp-1*^-/-^/*Ptc1*^+/-^ and *Ptc1*^+/-^ mice, respectively; *P* = 0.0038) or in *Ptc1*^+/-^ mice lacking *Rad54* (*i.e.,* the viable genotypes: *Rad54*^+/-^/*Parp-1*^-/-^/*Ptc1*^+/-^ and *Rad54*^-/-^/*Ptc1*^+/-^, 44% *vs* 15%, respectively; *P* = 0.0274) (Figure 1D-1F). Irradiation of *Ptc1*^+/-^ (*Ptc1*^+/-^) or *Rad54*^-/-^ mice (*Rad54*^-/-^/*Ptc1*^+/-^) caused significantly increase MB incidence compared with unirradiated mice, both in *wt Parp-1* background [*Ptc1*^+/-^ (82% vs 14%; *P* = 0.0001); *Rad54*^-/-^/*Ptc1*^+/-^ (84% vs 15%; *P* = 0.0001)] and in *Parp-1* heterozygotes (*Parp-1*^+/-^/*Ptc1*^+/-^ 56% vs 30%; *P* = 0.0447) (Figure 1D-1F). In contrast, irradiation of *Parp-1*^-/-^/*Ptc1*^+/-^ mice caused a non-statistical increase of their already high MB incidence (75% vs 50%; *P* = 0.1717). This difference became significant only in mice with concurrent inactivation of one copy of *Rad54* [(*Rad54*^+/-^/*Parp-1*^-/-^/*Ptc1*^+/-^ mice) (79% vs 44%; *P* = 0.0378)] (Figure 1E and 1F). Notably, in a null *Rad54* background deletion of one *Parp-1* allele caused significantly lower radiation-induced MB tumorigenesis compared with *Parp-1 wt* mice [*Rad54*^-/-^/*Parp-1*^+/-^/*Ptc1*^+/-^ (56%) *vs Rad54*^-/-^/*Ptc1*^+/-^ mice (84%), *P* = 0.0439] (Figure 1E and 1F), suggesting a lethal haploinsufficient interaction between *Rad54* and *Parp-1* in the context of radiation oncogenesis. In all groups, irradiation increased the incidence of MB, an early-onset tumor. This was reflected in decreased incidence of late occurring tumors, including sarcomas and other tumors (Figure 1F), with exception of *Parp-1*^-/-^/*Ptc1*^+/-^ mice, in which MB mortality was already high and did not significantly increase with irradiation.

Loss of the normal *Ptc1* allele is the causative event in MB development in *Ptc1*^+/-^ mice [24]. To test if single or combined *Rad54* and *Parp-1* deficiency alters the typical chr-13 LOH pattern in MB, we performed microsatellite analysis with a set of markers spanning the length of chr-13, where *Ptc1* gene is located (Figure S1). Within the sample size, no obvious differences were observed in *Ptc1* LOH pattern in single or compound *Rad54* and *Parp-1* mutants.

### Combined inactivation of *Rad54* and *Parp-1* causes growth retardation and perinatal lethality

**Figure 2:**
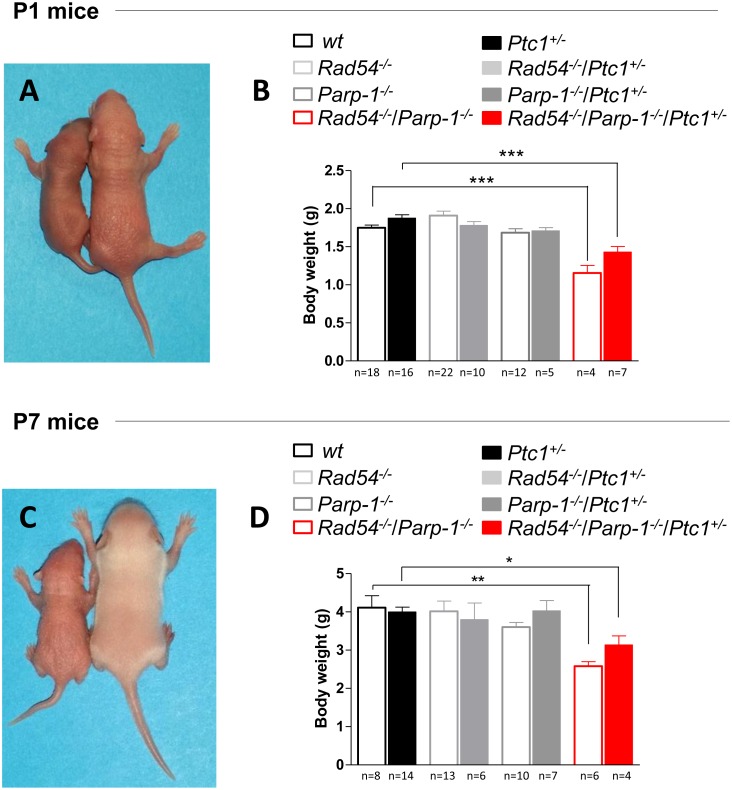
Effect of combined inactivation of ***Rad54*** and ***Parp-1*** on mouse development Left in **(A)** and **(C)** *Rad54*^-/-^/*Parp-1*^-/-^ mice exhibited reduced size compared to *wt* and *Ptc1*^+/-^ littermates (right) at **(A)** postnatal day 1 and **(C)** 7. Double knockout *Rad54*^-/-^/*Parp-1*^-/-^ mice showed significantly reduced body weight at **(B)** postnatal day 1 and **(D)** 7 compared *wt* and *Ptc1*^+/-^ littermates, demonstrating that combined loss of *Rad54* and *Parp-1* causes severe growth retardation. The number of mice used per test is indicated in the graph (n). *P ≤ 0.05; **P ≤ 0.005; *** *P* ≤ 0.0001.

The complete lack of *Rad54*^-/-^*/Parp-1*^-/-^ null mice in our triple cross at weaning age prompted us to investigate the effect of this genetic interaction at earlier developmental times. Analysis of the genotype distribution in the offspring at postnatal day 1 (P1) revealed that, of 764 newborns derived from heterozygous mating, only 10 and 13 in *wt* and *Ptc1*^+/-^ genetic backgrounds, respectively, were *Rad54*^-/-^/*Parp-1*^-/-^ compound mutants, far below the 24 mice expected based on a Mendelian inheritance ratio of 25%, although this trend reached statistical significance only in *Ptc1*^+/+^ mice (Table 1). In addition, double knockout *Rad54*^-/-^/*Parp-1*^-/-^ pups at P1 and P7 showed significantly smaller body size compared to relative littermates (Figure 2A and 2C). In fact, inactivation of both genes significantly reduced body weight at P1 (by 34% in *wt* and 17% in *Ptc1*^+/-^ genotypes) and P7 (by 37% in *wt* and 20% in *Ptc1*^+/-^ genotypes), showing that combined loss of *Rad54* and *Parp-1* causes severe growth retardation (Figure 2A-2D). Moreover, 45% and 29% of the double mutants on *Ptc1*^+/-^ and *wt* background, respectively, dyed during the first fortnight of life and none survived to weaning age (data not shown).

**Table 1:** Genotype distribution in the offspring of the intercross *Rad54*^+/-^/*Parp-1*^+/-^/*Ptc1*^+/+^ x Rad54^+/-^/*Parp-1*^+/-^/*Ptc1*^+/-^ at birth

Genotype			Expected	Observed (%)
*Rad54*	*Parp-1*	*Ptc1*		Total mice = 764
+/+	+/+	+/+	24	17 (71)
+/+	+/+	+/-	24	26 (109)
-/-	+/+	+/+	24	29 (121)
-/-	+/+	+/-	24	21 (88)
+/+	-/-	+/+	24	20 (84)
+/+	-/-	+/-	24	14 (58)
-/-	-/-	+/+	24	10 (41) *
-/-	-/-	+/-	24	13 (54) ^1^

The table represents expected and observed genotype distribution in the offspring at birth. Compound mutants were born at numbers below the predicted Mendelian inheritance ratio. Mice heterozygous for *Rad54* and/or *Parp-1* are not shown. * *P* ≤ 0,05; ^1^*P* = 0,0948 Fisher's exact test.

### Effects of combined inactivation of *Rad54* and *Parp-1* on cerebellum morphogenesis

**Figure 3:**
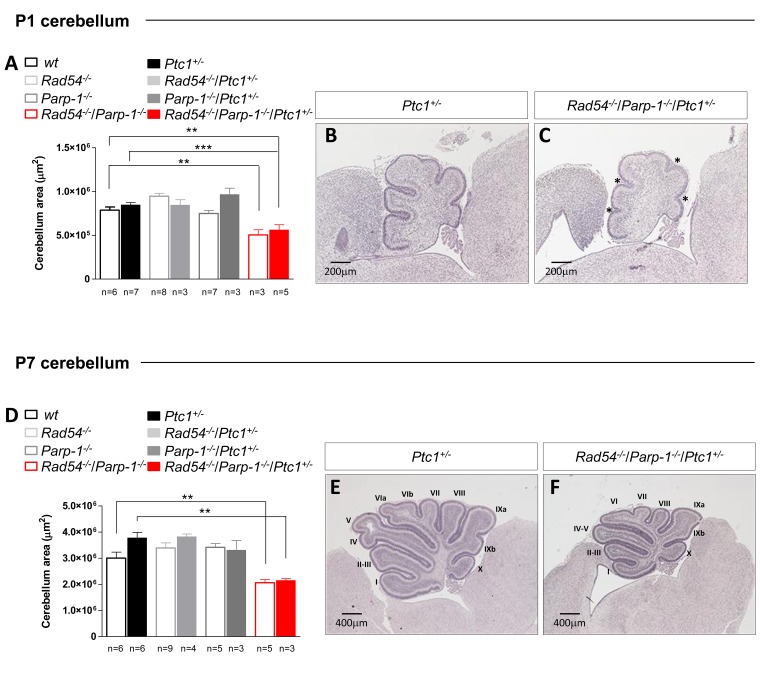
Effect of combined inactivation of ***Rad54*** and ***Parp-1*** in the developing cerebellum Rad54^-/-^/*Parp-1*^-/-^ double mutant mice show significant reduction of cerebellum area compared to *wt* and *Ptc1*^+/-^ mice both at **(A)** P1 and **(D)** P7. **(B** and **E)** **H** & **E** stained sagittal sections of *Ptc1*^+/-^ and **(C** and **F)** *Rad54*^-/-^/*Parp-1*^-/-^/*Ptc1*^+/-^ cerebellum at P1 and P7. The number of mice used per test is indicated in the graph (n). *P ≤ 0.05; **P ≤ 0.005; *** *P* ≤ 0.0001.

To further clarify the underlying mechanisms of synthetic lethal interactions of *Rad54* and *Parp-1* perturbation, we focused on the cerebellum, from which MB originates. The well-characterized developmental processes and stereotypical foliation pattern make this organ particularly amenable to the study of factors affecting development, leading to abnormal morphology or function. By measuring the cross-sectional area of developing cerebellum at P1 and P7 we show that, in line with generalized growth delay, significant decrease in size was observed in cerebella from *Rad54*^-/-^/*Parp-1*^-/-^ mutants at P1 and P7 relative to *Ptc1*^+/+^ mice (by 36% at P1, *P* = 0.0035, and 31% at P7, *P* = 0.0078; Figure 3). Similarly decreased cerebellum area was observed in double null mice with *Ptc1*^+/-^ genotype (by 34% at P1, *P* = 0.0007 and 43% at P7, *P* = 0.0012) (Figure 3A-3F). In keeping with growth delay, cerebella from *Rad54*^-/-^/*Parp-1*^-/-^/*Ptc1*^+/-^ mice showed shallow principal fissures at P1 relative to *Ptc1*^+/-^ mice (asterisks in Figure 3C). In addition, delayed lobularization was evident at P7 (Figure 3E and 3F).

### Proliferation, DNA damage and cell death in CGNPs of cerebellum in *Rad54* and *Parp-1* double mutants

**Figure 4:**
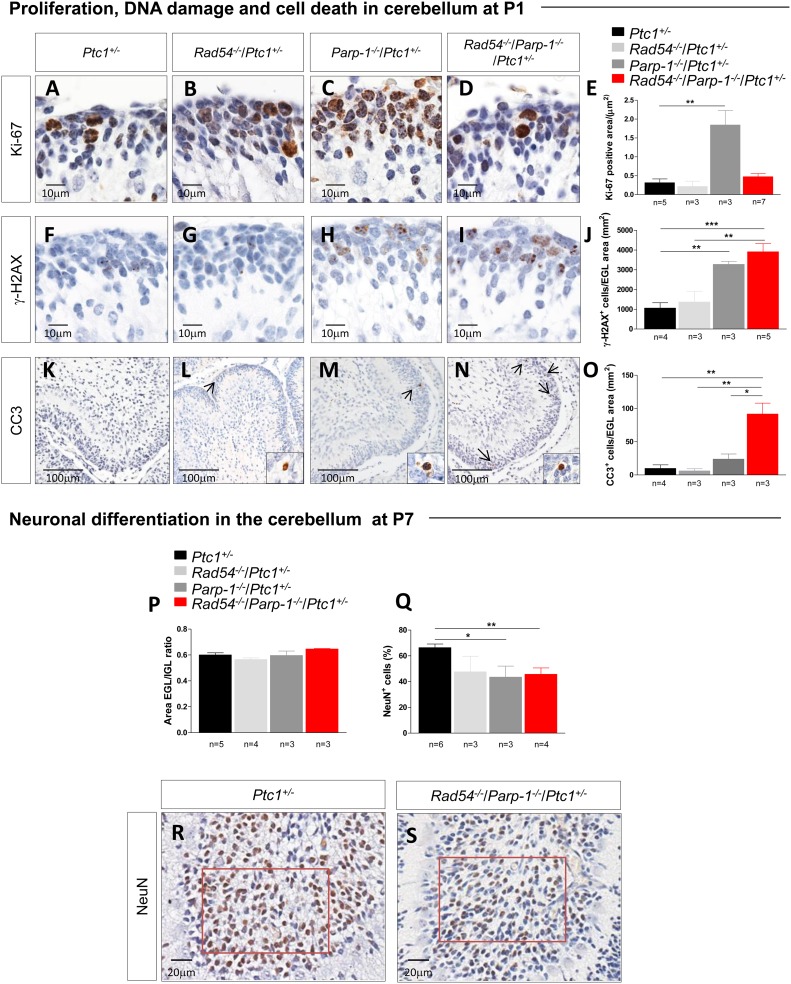
Evaluation of proliferation, DNA damage and cell death in neuronal precursors of the EGL at P1 and differentiation in mature granule neurons in the IGL at P7 **(A**-**E)** Immunohistochemical analyses for Ki-67 showing significant increased expression in *Parp-1*^-/-^/*Ptc1*^+/-^ compared to *Ptc1*^+/-^ mice. **(F**-**J)** Immunohistochemical analyses for γ-H2AX showing significantly increased expression in *Parp-1*^-/-^/*Ptc1*^+/-^ and *Rad54*^-/-^/*Parp-1*^-/-^/*Ptc1*^+/-^ compared to *Ptc1*^+/-^ mice. **(K**-**O)** Immunohistochemical analyses for cleaved caspase-3 (CC3) showing significantly increased expression in *Rad54*^-/-^/*Parp-1*^-/-^/*Ptc1*^+/-^ mice compared to *Rad54*^-/-^/*Ptc1*^+/-^*, Parp-1*^-/-^/*Ptc1*^+/-^ or *Ptc1*^+/-^ mutant mice. **(P)** The ratio between EGL and IGL area was similar in mice of all genotypes. **(Q)** Significant decrease in number of mature neurons expressing NeuN in *Parp-1*^-/-^/*Ptc1*^+/-^ and *Rad54*^-/-^/*Parp-1*^-/-^/*Ptc1*^+/-^ mutants compared to *Ptc1*^+/-^ mice. **(R** and **S)** Expression level of NeuN was determined in a fixed area (in red) from folia VIII. The number of mice used per test is indicated in the graph (n). *P ≤ 0.05; **P ≤ 0.005; *** *P* ≤ 0.0001.

We next analyzed the cellular mechanisms underlying synthetic lethal interactions between *Rad54* and *Parp-1* by focusing on the cerebellum of *Ptc1*^+/-^ mutants. The neonatal cerebellum undergoes rapid postnatal development, occurring through a burst of proliferation of granule cell precursors (GCPs) in the external granule layer (EGL) on the cerebellar surface. Notably, highly proliferating cells are particularly susceptible to endogenous and exogenous DNA damage, due to the presence of numerous replication forks that may stall and eventually undergo DSBs after collision with DNA damage [25,26]. This makes this cellular population suitable for investigating DNA repair-related mechanisms responsible for the growth delay observed. Quantification of Ki-67 staining in the EGL revealed a high proliferation rate in *Parp-1*^-/-^/*Ptc1*^+/-^ mice (Figure 4A-4E), which is in keeping with the higher spontaneous MB tumorigenesis detected in *Parp-1*^-/-^ mice. Similarly high growth rates were not observed in *Rad54*^-/-^/*Parp-1*^-/-^/*Ptc1*^+/-^ mice, suggesting that persistent SSBs formation due to *Parp-*1 inhibition, promoting collapse of replication forks and DSBs which would normally be repaired by HR, cannot be repaired effectively in the absence of functional *Rad54*.

We therefore evaluated whether concomitant inactivation of *Rad54* and *Parp-1* enhances DNA damage and apoptosis in the neural precursor population of the EGL of double- compared to single-mutants or *wt* mice. To this aim, P1 brains were immunostained with a DSB (γ-H2AX), or an apoptotic marker (cleaved caspase-3), and the staining in the EGL was quantified. Consistent with its recognized role in resolution of SSB and stalled replication forks, inactivation of *Parp-1*, but not *Rad54*, resulted in a significant 3-fold increase of spontaneous DSBs compared to *Ptc1*^+/-^ mice (*P* = 0.0013). A 4-fold increase (*P* = 0.0010) was also detected in *Rad54*^-/-^*/Parp-1*^-/-^/*Ptc1*^+/-^ mutants (Figure 4F-4J). However, despite similar amount of DSBs induced by inactivation of *Parp-1* and by combined inactivation of *Parp-1* and *Rad54*, we detected large differences in positivity for cleaved caspase-3 among the different mutants (Figure 4K-4O). Mice with combined inactivation of *Rad54* and *Parp-1* (*Rad54*^-/-^*/Parp-1*^-/-^*/Ptc1*^+/-^) showed significantly increased staining for cleaved caspase-3 expression compared to *Rad54*^-/-^ (*Rad54*^-/-^*/Ptc1*^+/-^*;* 15.3-fold, *P* = 0.007*), Parp-1*^-/-^ (*Parp-1*^-/-^*/Ptc1*^+/-^*;* 3.9-fold, *P* = 0.020) or *Ptc1*^+/-^ (*Ptc1*^+/-^*;* 9.3-fold, *P* = 0.003) mice. These findings confirm that *Parp-1* inactivation causes an excess of spontaneous DSBs in neural cells, which can be partly repaired if *Rad54* is active, but result in high apoptotic levels in GCPs with concurrent *Rad54* inactivation.

In the neonatal cerebellum, the proliferation phase is followed by differentiation processes, consisting in cell cycle exit and inward migration of postmitotic GCPs to the internal granule layer (IGL), which causes progressive EGL disappearance and formation of the IGL of mature granule neurons. To evaluate the effects of combined inactivation of *Rad54* and *Parp-1* on differentiation processes, we measured the areas of the EGL and IGL in the cerebellum of *Ptc1*^+/-^ mice with single or combined inactivation of *Rad54* or *Parp-1* at P7, and calculated their ratio. Despite drastic decrease in cerebellum size, no modification of the EGL/IGL ratio was detected in mice with combined inactivation of *Rad54* and *Parp-1* relative to other genotypes, indicating no overt alterations in the balance between proliferation (EGL) and differentiation (IGL) in the cerebellum caused by single or combined inactivation of the genes under investigation (Figure 4P). However, quantification of the density of neurons labeled by the NeuN marker of mature neurons in fixed regions of the IGL (crest number VIII, red square in Figure 4R and 4S), revealed a significant decrease in the density of NeuN+ mature neurons in *Parp-1*^-/-^*/Ptc1*^+/-^ (43.5%, *P* = 0.0126) and *Rad54*^-/-^*/Parp-1*^-/-^*/Ptc1*^+/-^ mice (45.7%, *P* = 0.0042), suggestive of depletion of granule neurons (Figure 4Q and 4S).

### Synthetic lethality affects the expression of DNA repair related gene

**Figure 5:**
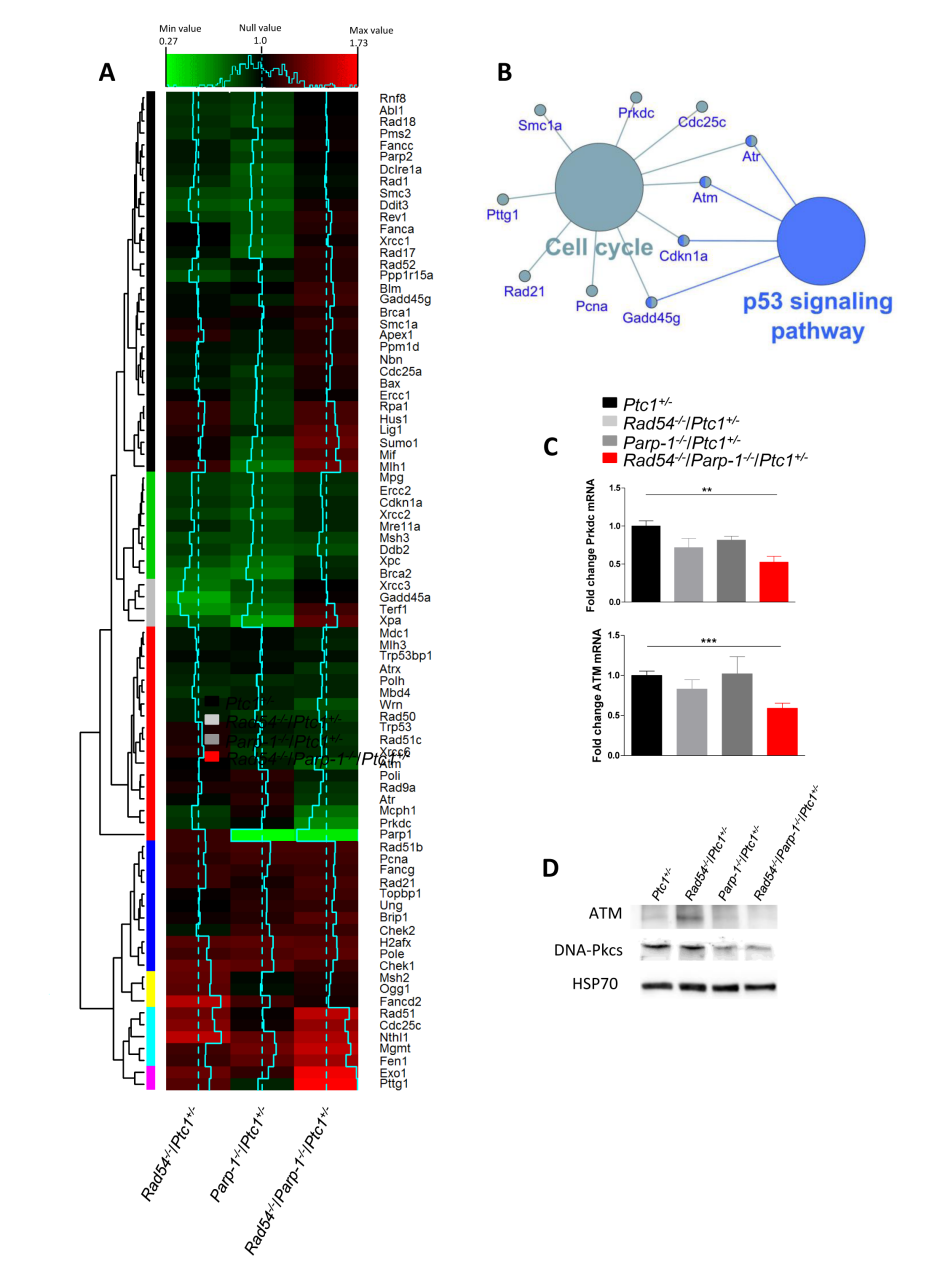
Bioinformatic analysis of expression profiles of DNA repair-related genes in P1 cerebella from ***Rad54***^-/-^**/*Ptc1***^+/-^, ***Parp-1***^-/-^**/*Ptc1***^+/-^, ***Rad54***^-/-^/***Parp-1***^-/-^/***Ptc1***^+/-^ **and *Ptc1***^+/-^ mice **(A)** Heat map, generated from RT^2^ Profiler PCR Arrays Mouse DNA Damage, representing the fold-change variation in the three genotypes (solid blue lines) versus the *Ptc1*^+/-^ mice (dotted blue lines). Relative hierarchical clustered groups represented by colored bars on left side. **(B)** Gene Ontology network generated by analysis of the most significantly perturbed KEGG pathways in the *Rad54*^-/-^/*Parp-1*^-/-^/*Ptc1*^+/-^ mice when compared to the *Ptc1*^+/-^ mice. **(C)** SYBR Green real-time PCR validation of *ATM* and *Prkdc* (*DNA-PKcs*) genes. **(D)** Immunoblot analysis of ATM and DNA-PKcs proteins with relative HSP70 as loading control. *P ≤ 0.05; **P ≤ 0.005; *** *P* ≤ 0.0001.

The robust synthetic lethal effect of combined *Rad54* and *Parp-1* inactivating mutations led us to investigate the underlying molecular mechanisms by addressing the modification of the expression profile of 84 genes with established roles in DNA damage signaling. We carried out this analysis in the P1 cerebellum of *Ptc1*^+/-^ mice with single or combined inactivation of *Rad54* and/or *Parp-1* (*Rad54*^-/-^/*Ptc1*^+/-^, *Parp-1*^-/-^*/Ptc1*^+/-^ and *Rad54*^-/-^/*Parp-1*^-/-^*/Ptc1*^+/-^) and results were compared with those from *Ptc1*^+/-^ mice using a pathway-based PCR expression array. In *Rad54*^-/-^/*Ptc1*^+/-^ mice 14 genes were significantly downregulated (16.7%) and 8 upregulated (9.5%); in *Parp-1*^-/-^*/Ptc1*^+/-^ mice, 22 genes were significantly downregulated (26%) and 6 (7%) upregulated; in *Rad54*^-/-^/*Parp-1*^-/-^*/Ptc1*^+/-^ mice, 10 genes (12%) were downregulated and 16 (19%) upregulated (S1 Table). The magnitude of many of these changes was small. However, as DNA repair genes are expressed at basal level in mammals, detection of small changes does not necessarily mean a negligible biological significance, but rather that even slight deregulation may significantly modify DNA repair capacity. We also performed a hierarchical clustering analysis that produced different groups of genes (colored bars, left side) based on similar fold-change patterns among the three genotypes (*Rad54*^-/-^/*Ptc1*^+/-^*, Parp-1*^-/-^*/Ptc1*^+/-^, *Rad54*^-/-^/*Parp-1*^-/-^*/Ptc1*^+/-^ mice) (Figure 5A). In particular, the violet module (lowest bar) shows the most upregulated genes in *Rad54*^-/-^/*Parp-1*^-/-^*/Ptc1*^+/-^ mice (i.e., *Exo1*, *P* = 0.031; *Pttg1*, *P* = 0.001); in the gray module are listed the genes downregulated in *Rad54*^-/-^/*Ptc1*^+/-^ and *Parp-1*^-/-^*/Ptc1*^+/-^ mutants that are upregulated in *Rad54*^-/-^*/Parp-1*^-/-^*/Ptc1*^+/-^ mice, of which *Xpa* was statistically significant (*P* = 0.033); instead, in the red module, the synergistic effects of combined *Rad54* and *Parp-1* mutations lead to downregulation of several genes (i.e., *Atm, P =* 0.031; *Atr, P =* 0.008*; Atrx, P =* 0.006; *Parp-1*, *P =* 0.001; *Prkdc*, *P =* 0.029; *Rad50*, *P =* 0.028; *Wrn*, *P =* 0.016) (Figure 5A and Table S1).

Pathway analysis of all significantly deregulated genes was performed using SPIA algorithm. The signaling pathway showing a significant perturbation in *Rad54*^-/-^*/Parp-1*^-/-^*/Ptc1*^+/-^ mice was the “Cell Cycle” pathway, including 10 genes (i.e. *Atm, Cdc25c, Cdkn1a, Pcna, Prkdc, Rad21, Gadd45g, Smc1a, Atr, Pttg1)* with fold changes and topological distribution suggesting pathway inhibition. Further analysis of these genes, performed with the Cytoscape gene ontology tool Cluego, showed a substantial overlap with the p53 signaling pathway (Figure 5B).

We validated *Atm* and *Prkdc* (*DNA-PKcs*), by RT-PCR confirming a significantly decreased expression in *Rad54* and *Parp-1* compound mutants (*Rad54*^-/-^/*Parp-1*^-/-^/*Ptc1*^+/-^) compared to single mutants (Figure 5C) and verified the decreased expression levels of ATM and DNA-PKcs by immunoblotting (Figure 5D).

### p53/p21-mediated senescence as underlying mechanism for the lethal phenotype of *Rad54*^-/-^/*Parp-1*^-/-^ compound mutants

**Figure 6:**
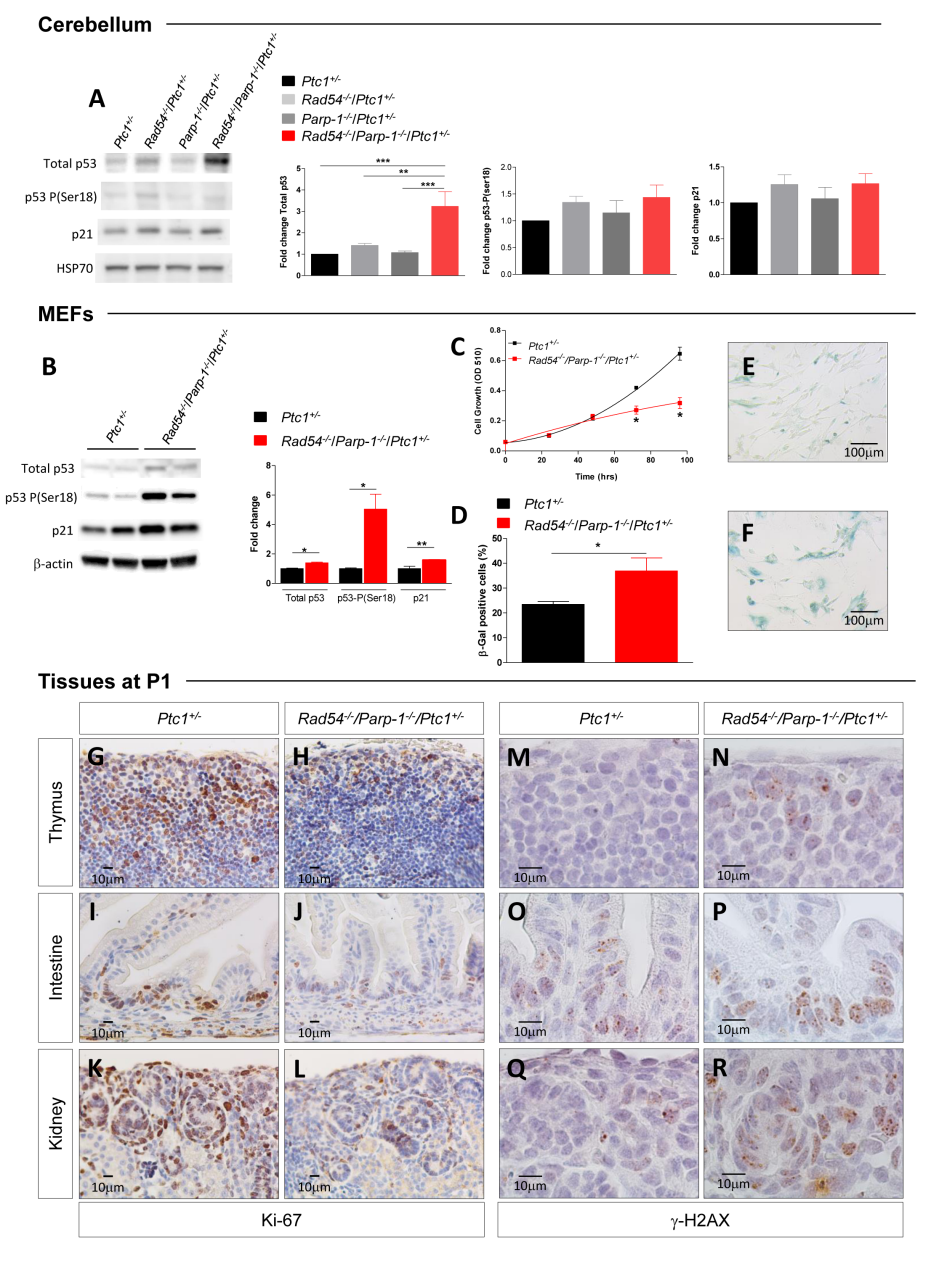
Evaluation of biomarkers of senescence **(A)** Immunoblot and densitometric analysis of p53/p21 signaling [total p53, p53-P(ser18) and p21] in cerebellum of *Ptc1*^+/-^*, Rad54*^-/-^/*Ptc1*^+/-^, *Parp-1*^-/-^/*Ptc1*^+/-^, and *Rad54*^-/-^/*Parp-1*^-/-^/*Ptc1*^+/-^ mice at P1, and **(B)** in MEFs from *Ptc1*^+/-^ and *Rad54*^-/-^/*Parp-1*^-/-^/*Ptc1*^+/-^ mice (two independent cell lines per genotype); β-actin and HSP70 were used as loading controls. **(C)** Growth kinetics and **(D)** quantification of SA-β-GAL staining in MEFs from *Ptc1*^+/-^ and *Rad54*^-/-^/*Parp-1*^-/-^/*Ptc1*^+/-^ mouse embryos. **(E)** Representative images of SA-β-GAL staining in MEFs from *Ptc1*^+/-^, and **(F)** and *Rad54*^-/-^/*Parp-1*^-/-^/*Ptc1*^+/-^ mouse embryos. **(G**-**L)** Proliferation (Ki67) and **(M**-**R)** DNA damage (γ-H2AX) in different tissues from *Ptc1*^+/-^ and *Rad54*^-/-^/*Parp-1*^-/-^/*Ptc1*^+/-^ neonatal mice at P1. The number of mice used per test is indicated in the graph (n). *P ≤ 0.05; **P ≤ 0.005; *** *P* ≤ 0.0001.

Based on results of bioinformatic analysis, we analyzed p53 expression in protein extracts from P1-cerebellum by immunoblotting. Although no significant differences were detected between *Rad54* and *Parp-1* single and compound mutants in phosphorylation of p53 at Ser18 [27], we observed a significant increase in total p53 expression (3-fold) only in compound mutants, indicating marked p53 activation (Figure 6A), which is consistent with persistent genetic damage. We also show that p53 stabilization did not occur at transcriptional level, as no changes in the expression of p53 were detected in the array (Figure 5A and Table S1), suggesting, instead, posttranslational modification. Immunoblotting for p21 showed a trend towards increased expression that, however, was not significant (Figure 6A).

To evaluate the effect of combined *Rad54* and *Parp-1* mutations at organismal level, we established mouse embryonic fibroblasts (MEFs) from *Rad54*^-/-^/*Parp-1*^-/-^/*Ptc1*^+/-^ and *Ptc1*^+/-^ mice. Immunoblot analysis of p53/p21 signaling pathway in MEFs of different genotypes showed significantly increased expression of p53-P(ser18), p53 and p21 in *Rad54*^-/-^/*Parp-1*^-/-^/*Ptc1*^+/-^ mutants compared to *Ptc1*^+/-^ MEFs (Figure 6B). Moreover, MEFs from *Rad54*^-/-^/*Parp-1*^-/-^/*Ptc1*^+/-^ mice showed decreased cell growth potential compared to those from *Ptc1*^+/-^ mice. The difference in growth rate become significant at 72 and 96 h after seeding, with a 1.5-2 fold reduction in cell numbers and an increase in doubling times (23 *vs* 35 h) in MEFs from *Rad54*^-/-^/*Parp-1*^-/-^/*Ptc1*^+/-^ compared to those from *Ptc1*^+/-^ mice (Figure 6C). We also detected a significantly greater fraction of positive cells after staining with Senescence-Associated β-Galactosidase (SA-β-GAL) (1.6-fold; *P* = 0.048), a biomarker of senescent cells, accompanied by senescence-associated morphological cellular changes (i.e., enlargement and flattening) (Figure 6E and 6F), suggesting accelerated acquisition of senescence in *Rad54*^-/-^/*Parp-1*^-/-^/*Ptc1*^+/-^ compared to *Ptc1*^+/-^ MEFs.

Although identification of senescent cells is challenging, especially *in vivo*, loss of replicative markers such as Ki-67 and sustained DNA damage are often considered as *bona fide* markers of senescence. We immunostained several tissues (thymus, lung, kidney, liver, intestine, skin) from *Rad54*^-/-^*/Parp-1*^-/-^*/Ptc1*^+/-^ and *Ptc1*^+/-^ mice at P1 with Ki-67 and γ-H2AX. Compared to *Ptc1*^+/-^ mice, we observed a general decrease in proliferation and increase in DNA damage in all tissues from *Rad54*^-/-^/*Parp-1*^-/-^/*Ptc1*^+/-^ mice, particularly in thymus, intestine and kidneys (Figure 6G-6R). Instead, immunostaining with caspase-3 did not reveal obvious differences in apoptosis (data not shown). Altogether, these data strongly support the involvement of p53/p21-mediated accelerated senescence in growth delay of *Rad54*^-/-^/*Parp-1*^-/-^ compound mutants, culminating in a lethal perinatal phenotype.

## DISCUSSION

The DNA repair system functions as a critical tumor suppressor network to preserve the integrity of the genome preventing malignancy, and DNA repair defects are frequently encountered in human cancer. Therefore, gaining insights into the mechanism of action of DNA repair genes is pivotal to development of novel tumor-specific treatment strategies targeting deregulated DNA repair [28]. A major obstacle to achieving synthetic lethal therapies is a lack of insight into the phenomenon in cancer cells, and insufficient knowledge of the molecular mechanisms influencing synthetic lethal interactions.

We earlier investigated *in vivo* genetic interactions between *Parp-1* and *Ptc1*, and found that combined inactivation of the two genes is not critically important for normal development and survival of the compound mutant mice; however, increased genetic instability caused by abrogation of *Parp-1* was able to promote MB formation in *Ptc1*^+/-^ mice by fostering accumulation of genetic defects and facilitation of loss of the remaining normal *Ptc1* allele [22]. Despite the critical role of the HR system for embryonic development, attenuated HR by deletion of *Rad54* also did not result in lethality in *Ptc1*^+/-^ mice, implying redundancy in function of proteins involved in HR; however, in previous work, by exposing double *Rad54/Ptc1* mutants to 2 Gy, we detected significantly increased MB tumorigenesis, possibly through the generation of viable but genetically rearranged neural cell precursors due to repair of DNA lesions by error-prone mutagenic pathways [21]. An analogous increase was not seen here, likely due to the lower dose used and relative radioresistance of *Rad54*^-/-^ mice [23].

Former *in vitro* studies have shown that *Parp-1* inhibitors induce synthetic lethality in cells that are defective in HR [1,2,29]. An extensive body of literature has since documented the impact of Parp inhibitors on synthetic lethality in cells that are defective in HR due to loss of HR-associated proteins, and their use as single-agent therapies acting through the concept of synthetic lethality [30]. The results shown here demonstrate for the first time that *Parp-1* and *Rad54* are genetically linked and that they share overlapping functions that are essential in mouse development. We observed not only that the combination is active *in vivo*, but could also clarify that *Parp-1* is not strictly necessary for HR during early embryogenesis, as combined deletions of *Parp-1* and *Rad54* caused significantly reduced birth rate and growth retardation - resulting in early death of *Rad54*^-/-^*/Parp-1*^-/-^ pups - not full embryonic lethality.

Since evaluation of the effects of simultaneous *Rad54* and *Parp-1* inactivation on MB development was prevented by the lack of viable compound mutants, we investigated the inactivation of DNA repair genes in the context of *Ptc1* tumor suppression by examining mice with complete abrogation of either *Rad54* or *Parp-1* function, in combination with heterozygosis of *Parp-1* or *Rad54*, respectively. Attenuation of *Rad54* in the context of *Parp-1* abrogation had synthetic lethal effects in dosage-independent manner. In contrast, inactivation of one copy of *Parp-1* in the setting of *Rad54* abrogation had two distinct outcomes depending on treatment, *i.e.,* no effect on spontaneous tumor development, or decreased induction of MB induced by radiation. A further increase in *Parp-1* inactivation (*i.e.*, both copies) caused a shift to synthetic lethality (*i.e.*, loss of viable progeny), demonstrating that combined defects in *Rad54* and *Parp-1* cause synthetic lethal interactions in a *Parp-1*-gene dosage dependent manner, and highlighting the delicate balance that must be hit when targeting essential pathways.

Important to our understanding of the underlying mechanisms of this novel genetic link, immunohistochemical analysis revealed a trend to high degree of granule cell proliferation in the EGL of *Parp-1* null mice (*Parp-1*^-/-^*/Ptc1*^+/-^), which decreased significantly in age-matched double mutants (*Rad54*^-/-^*/Parp-1*^-/-^*/Ptc1*^+/-^). This was accompanied by substantial delay in neuronal differentiation, as shown by sparse NeuN staining and limited presence of committed GCPs in both groups. Given the concurrent high levels of spontaneous DSBs in both *Parp-1* null mutants (*Parp-1*^-/-^*/Ptc1*^+/-^ and *Rad54*^-/-^*/Parp-1*^-/-^*/Ptc1*^+/-^), the low apoptotic levels observed only in *Parp-1*^-/-^*/Ptc1*^+/-^ correlate well with the high spontaneous rate of MB in these mice, and suggest that the cells can survive with a mutation in either repair pathways, not both, which causes cells to die.

As several DNA repair deficiency syndromes (*e.g.* ataxia telangiectasia, and spinocerebellar ataxia with axonal neuropathy1) target the cerebellum [31], we set up to investigate how alternative repair pathways compensate the impairment of predominant DNA repair mechanisms to maintain cerebellum integrity. We compared gene expression changes in DNA repair-related genes in *Rad54* and *Parp-1* single (*Rad54*^-/-^*/Ptc1*^+/-^*; Parp-1*^-/-^/*Ptc1*^+/-^) and combined mutants (*Rad54*^-/-^*/Parp-1*^-/-^/*Ptc1*^+/-^) *versus Ptc1*^+/-^ mice. We detected strong downregulation of *Prkdc* (*DNA-PKcs*), which was unexpected as *DNA-PKcs* activation has been invoked to explain the synthetic lethal effects of *Parp-1* inhibitors in HR-defective cells [32]. *DNA-PKcs* downregulation was paralleled by marked downregulation of *Atm*. *DNA-PKcs* and *Atm* are both members of the phosphatidylinositol 3-kinase (PI3K)-related protein kinase superfamily and act in tandem by important cross-talk [33,34]. Notably, combined loss of *Atm* and *DNA-PKcs* cause embryonic lethality in mice [35,36], suggesting that their downregulation may be central to the perinatal lethality of *Rad54*^-/-^*/Parp-1*^-/-^ mutants. PI3K and Parp-1 inhibitors has proven effective in treating Brca1-related breast cancer *in vivo* [37].

Driven by the results of the bioinformatic analysis converging on p53 signaling, we investigated the expression of p53 protein in P1-cerebellum and MEFs, detecting significant accumulation of p53 protein in both. Because p53 phosphorylation is classically considered as the first crucial step of p53 stabilization, driven by several kinases, including ATM and DNA-PKcs, we analyzed phosphorylation of p53 at Ser18 [27] in P1 cerebella and did not detect significant differences among genotypes, despite accumulation of p53 only in double mutants. However, it is known that multiple phosphorylation sites exist, acting cooperatively for stimulating p53 activity [38]. Also, phosphorylation may not be a universal requirement for p53 stabilization, especially *in vivo*, as several studies have established, shifting attention on Mdm2 regulation of p53 [39]. All these factors, along with differences between *in vitro* and *in vivo* conditions, may concur to explain the differences in the expression pattern of p53/p53 Ser18 observed between MEFs and cerebellum.

Our results of p53 expression in P1-cerebellum and MEFs are in line with the fact that deficiency in several DNA repair genes such as *DNA Lig4*, *XRCC4* and *Rad51* cause increased level of genotoxic stress, triggering a p53-dependent DNA damage response leading to embryonic lethality [40–42]. More generally, mutations in over 150 cell-essential genes from different pathways result in recurrent phenotypes, which involve neural apoptosis and developmental delay in zebrafish and share p53 upregulation, suggesting that p53 might function as a developmental checkpoint triggered by various stressors, including genetic defects [43]. In agreement, mice with constitutively active p53 are generally not viable, due to inhibition of proliferation - *via* cell-cycle arrest, quiescence, senescence and differentiation - and/or induction of apoptosis [44]. The nature and intensity of damage, as well as, the level of p53 expression and its interaction with specific proteins, may determine the choice among the different cellular responses that overall are cell-type specific. Besides p53-dependent neuronal apoptosis, our findings that MEFs from compound *Rad54*/*Parp-1* mutant mice display severe p53-mediated accelerated senescent phenotypes, and that features of senescence were detected in several neonatal tissues, strongly argue for senescence as the mechanism underlying synthetic lethal interactions of *Rad54* and *Parp-1* inactivation *in vivo*. Notably, many of the DNA repair pathways found deregulated in the cerebellum of *Rad54*^-/-^/*Parp-1*^-/-^/*Ptc1*^+/-^ mutants have been reported to cause senescence; overexpression of *Pttg1*, the most upregulated gene in our settings, induces senescence in a p53-dependent fashion in normal fibroblasts [45], and chemical inhibition of *DNA-PKcs* leads to p53-dependent accelerated senescence after irradiation *in vivo* and *in vitro* [46].

In summary, our data contribute to the understanding of the interplay of *Rad54* and *Parp-1 in vivo* by showing that their combined genetic disruption caused a marked growth delay culminating in a perinatal lethal phenotype. We identified accumulation of DNA damage and enhanced apoptotic cell death as a driving mechanism for growth retardation during development of the cerebellum in *Rad54*/*Parp-1* compound mutants. Mechanistically, NHEJ was selectively repressed through downregulation of *DNA-PKcs*. Concomitant downregulation of *Atm* was also identified, directly involving transcriptional repression of PI3K signaling in synthetic lethality caused by *Rad54* and *Parp-1* deficiency. Finally, combined inactivation of *Rad54* and *Parp-1* caused accumulation of p53 and p21, which resulted in enhanced senescence in MEFs and several mouse tissues from *Rad54*^-/-^/*Parp-1*^-/-^/*Ptc1*^+/-^ mutants. The p53-depedent apoptotic/senescent phenotypes is also potentially tumor protective; in fact, inactivation of one copy of *Parp-1* in *Rad54*^-/-^/*Ptc1*^+/-^ mutants suppressed radiation-induced MB tumorigenesis, identifying haplosufficient interactions also during neoplastic growth. Investigating the functional interplay between the HR factor *Rad54* and *Parp-1 in vivo* during development and oncogenesis may help delineate the molecular mechanisms of lethal synthetic interactions and guide more successful clinical translation.

## MATERIAL AND METHODS

### Animal breeding

Mice lacking one *Ptc1* allele (*Ptc1*^neo6-7/+^, named *Ptc1*^+/-^ throughout the text) were generated through disruption of exons 6 and 7 in 129/Sv embryonic stem cells [18], and maintained on CD1 background. Genotyping for *Ptc1* was performed as described [18]. *Ptc1*^+/-^ mice were crossed with *Rad54*^-/-^ or *Parp-1*^-/-^ mice on C57Bl/6 background [47,48,49], to produce an F1. F1 mice of selected genotypes, i.e. *Rad54*^+/-^/*Ptc1*^+/-^ x *Parp-1*^+/-^/*Ptc1*^+/+^ and *Rad54*^+/-^/*Ptc1*^+/+^ x *Parp-1*^+/-^/*Ptc1*^+/-^ were crossed to produce an F2 population. Finally *Rad54*^+/-^/*Parp-1*^+/-^*/Ptc1*^+/+^/ and *Rad54*^+/-^/*Parp-1*^+/-^*/Ptc1*^+/-^ mice were intercrossed. Genotyping of mice was performed as described [21,49].

### Animal treatment and irradiation

Mice were housed under conventional conditions with food and water available *ad libitum* and a 12-h light cycle. For long-term study, mice were irradiated at postnatal day 1 (P1) with 1 Gy of X rays. Irradiation was performed using a Gilardoni CHF 320 G X-ray generator (Gilardoni S.p.A., Mandello del Lario, Italy) operated at 250 kVp, 5mA, Half-Value Layer = 1.6 mm Cu (additional filtration of 2.0 mm Al and 0.5 mm Cu) [50]. The size of the experimental groups was chosen to ensure statistical relevance. Randomized methods were applied and experiments were performed blinded. All experiments were conducted according to the directive 2010/63/EU of the European Parlament. Animal studies were approved and permission was issued by “Ministero della Salute” (Approval number is 986/2015-PR).

### Histological analysis and tumor quantification

Mice were observed daily for their lifespan. Upon decline of health (i.e., severe weight loss, paralysis, ruffling of fur, or inactivity), they were killed and autopsied. Normally appearing and tumor bearing brains were fixed in 10% buffered formalin or snap frozen in liquid nitrogen. Autopsied mice were also checked for the presence of any other tumor, and all tumor masses were collected and processed for histological analysis using standard methods. MB incidence was expressed as the percentage of mice with tumors.

### Tissue collection

Brains from *Ptc1*^+/-^, *Parp-1*^-/-^/*Ptc1*^+/-^, *Rad54*^-/-^/*Ptc1*^+/-^, and *Rad54*^-/-^/*Parp-1*^-/-^/*Ptc1*^+/-^ were collected at P1 and P7. Samples were fixed in 10% buffered formalin. For mRNA profiling and western blot analysis P1 cerebella were snap frozen in liquid nitrogen and stored at -80°C.

### Microsatellite analysis at the Ptc1 locus in MB

DNA from MBs and normal tissue was extracted using Wizard SV Genomic DNA Purification System (Promega Corporation, Madison, WI). Polymerase chain reaction amplifications were performed as described previously. About 10-14 microsatellites spanning the length of chromosome (chr) 13 were used to examine tumor DNA in comparison with the corresponding genomic DNA [51].

### Morphometric analysis

Cerebella external granule layer (EGL) and internal granule layer (IGL) total cross sectional areas were measured in at least 3 samples. The imaging software NIS-Elements BR 4.00.05 (Nikon, Instruments Europe B.V., Italy) was used for morphometric analyses.

### Immunohistochemical analysis

Cerebella and other tissues paraffin sections were used for immunohistochemical analysis as described previously [50]. Antibodies used were Ki-67 (ab15580, Abcam, Cambridge, UK), cleaved caspase-3 (9664, Cell Signaling Technology, Danvers, MA) and γ-H2AX (#2577, Cell Signaling Technology) and NeuN (MAB377, Millipore, Billerica, MA).

*Ki-67 quantification:* Three to 7 sections from EGL of *Ptc1*^+/-^, *Rad54*^-/-^/*Ptc1*^+/-^, *Parp-1*^-/-^/*Ptc1*^+/-^ and *Rad54*^-/-^/*Parp-1*^-/-^/*Ptc1*^+/-^ mice were immunostained for Ki67. Images were captured by HistoFAXS software (TissueGnostics GmbH, Vienna, Austria) at 20x magnification. Specific regions of interest (EGL) were analyzed with HistoQuest software (TissueGnostics) for automatic color separation and quantification. Expression levels were evaluated as stained area per μm^2^.

*γ-H2AX and cleaved-caspase-3 quantification:* Three to 5 sections from EGL of *Ptc1*^+/-^, *Rad54*^-/-^/*Ptc1*^+/-^, *Parp-1*^-/-^/*Ptc1*^+/-^ and *Rad54*^-/-^/*Parp-1*^-/-^/*Ptc1*^+/-^ mice were immunostained for γ-H2AX or cleaved caspase-3. expression levels were evaluated counting stained positive cells/ EGL area (mm^2^).

*NeuN quantification:* Three to 5 cerebellum sections of *Ptc1*^+/-^, *Parp-1*^-/-^/*Ptc1*^+/-^*, Rad54*^-/-^/*Ptc1*^+/-^ and *Rad54*^-/-^/*Parp-1*^-/-^/*Ptc1*^+/-^ mice were immunostained for NeuN. Expression levels of NeuN were evaluated as the percentage of stained positive cells in a fixed area (10000 μm^2^) of folia VIII for each cerebellum.

### Preparation of mouse embryonic fibroblast (MEFs)

MEFs (two independent embryos per genotype) were generated from embryos at gestational day 13.5 (E13.5) according to standard protocols [52]. Briefly, the whole embryo was disaggregated using a mechanical disaggregator system (Medimax, CTSV, Bruino, TO, Italy) and grown in DMEM with 15% fetal bovine serum (FBS), 100 units/ml penicillin, 100 mg/ml streptomycin, and 2mM L-glutammine, in a humidified incubator with 5% CO2. All experiments were performed with cells that had been subjected to 2 passages.

### Sulforhodamine B (SRB) assay

For the growth curve SRB assay was performed as described previously [53]. The plate was analysed at 510 nm optical density in the VICTOR X3 Multilabel Plate Reader (PerkinElmer, Waltham, MA). Doubling time (Dt) was calculated with the formula: Dt = t log2 / (log n1 – log n0), where n0 is the number of cell at time t0, n1 is the number of cells at time t1 and t is the time in exponential growth phase required by cells to increase their number from n0 to n1 (t = t1 – t0).

### SA-β-Gal staining

Cells were cultured for 24 h, and stained using the Senescence β-Galactosidase Staining Kit (Cell Signalling Technology). SA-β-Gal staining of MEFs was performed following manufacturer's instructions. The Percentage of cells expressing SA-β-Gal was quantified by inspecting at least 1000 cells in at least two different cell lines for each genotype.

### Immunoblotting

Appropriate amounts of proteins (30 μg or 60 μg), extracted from a pool of 3 P1-cerebella and MEFs were loaded and separated by SDS-PAGE. Proteins were electro transferred to PVDF or nitrocellulose membranes (Trans-Blot Turbo Transfer Pack, BIO-RAD Laboratories, Hercules, CA) at the Trans-Blot Turbo Transfer System (BIO-RAD Laboratories). After blocking, membranes were incubated over night at +4°C with primary antibodies against DNA-PKcs and p53 (ab70250 and ab31333, Abcam, Cambridge, UK), ATM, p21 (sc-23921 and sc-471, Santa Cruz Biotechnology, Santa Cruz, CA), anti-phospho-p53(Ser18) (9284, Cell Signaling Technology, Inc., Danvers, MA), β-actin and HSP70 (A5316 and H5147, Sigma-Aldrich, St Louis, MO). Membranes were probed for 1h at RT with appropriated HRP-conjugated secondary antibodies (Santa Cruz Biotechnology). Immunoreactive bands were visualized using Amersham ECL Prime WB detection reagent (GE Healthcare Europe, Milan, Italy). Images were acquired using a Image 6 quant LAS 500 (GE Healthcare Europe), and densitometric analysis was performed using ImageJ software.

### mRNA extraction and reverse transcription

Three to 7 cerebella were pooled for each genotype. mRNA was extracted with RNeasy Microarray Tissue kit (Qiagen, Germany) in accordance with manufacturer's instructions. Reverse transcription was accomplished with the RT2 First Strand kit (Qiagen, Germany) as suggested by manufacturer.

### RT2 Profiler PCR Array

RT^2^ RNA QC PCR Array (SaBiosciences, Qiagen, Germany) was used to test for RNA quality and inhibitors of RT-PCR analysis. For quantitative comparison of mRNA levels, real-time PCR was performed using RT^2^ Profiler PCR Arrays Mouse DNA Damage (SaBiosciences, Qiagen). For each genotype, two assays were carried out. Gene expression was related to the mean expression of all five housekeeping genes included in the array. Only Ct values <35 were included in the calculations. Data presented are averages of two independent experiments.

*Prkdc* and *Atm* genes were further validated by SYBR Green real-time PCR (SaBiosciences, Qiagen). The PCR primers for mouse *Atm* (PPM03454C-200 RT^2^ qPCR Primer Assay ) and *Prkdc* (PPM03711E-200 RT^2^ qPCR Primer Assay) were purchased from SABiosciences, Qiagen and real-time PCR were performed according to manufacturer's instructions. Data represent the average of three independent experiments.

### Bioinformatic analysis

Hierarchical clustering analysis was performed to evaluate similarities among the three genotypes when compared to *Ptc1*^+/-^mice. Euclidean distance matrices were performed in R (http://www.r-project.org/) using the fold change of each gene. Heat map visualization generated by R package “gplots” was used to show RT2 Profiler PCR Arrays Mouse DNA Damage. To estimate genotype-dependent pathway perturbations, the Signaling Protein Impact Analysis (SPIA) [54] algorithm based on KEGG (Kyoto Encyclopedia of Genes and Genomes) database was applied using the online tool Graphite (http://graphiteweb.bio.unipd.it/index.html). Significantly perturbed genes obtained from SPIA were analyzed by the Cytoscape tool [55] to evaluate the most perturbed pathways.

### Statistical analysis

Appropriate statistical tests were used. Analyses were performed using GraphPad Prism version 4.02 for Windows (GraphPad Software, San Diego, CA). Statistical comparisons were made using Student's *t*-test. Kaplan-Meier survival curves were compared and log rank test *P* values were calculated. *P* values ≤ 0.05 were considered statistically significant.

## SUPPLEMENTARY MATERIALS FIGURE AND TABLE




