# Supplementary material for: Synthetic lethal genetic interactions between Rad54 and PARP-1 in mouse development and oncogenesis
Source: Oncotarget. 2016 Jul 7;8(60):100958–74. doi: 10.18632/oncotarget.10479 (PMC5731848; doi:10.18632/oncotarget.10479)
Supplement: Supplementary file 1 [file oncotarget-08-100958-s001.pdf]

The association between MAD2 and prognosis in cancer: A systematic review and meta-analyses

Supplementary Materials

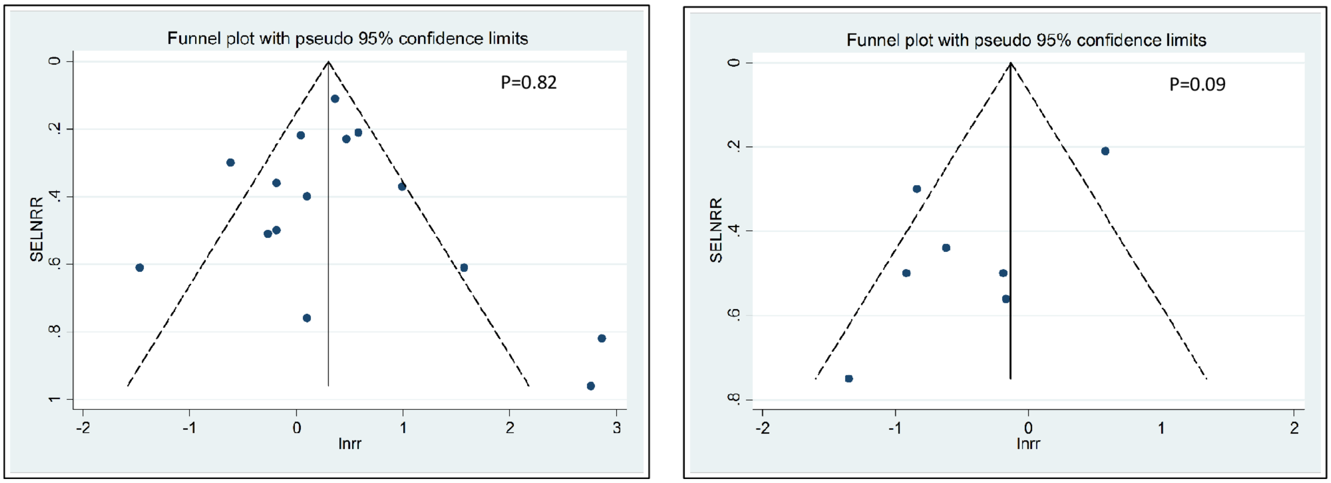

Supplementary Figure 1: Funnel plots of studies investigating MAD2 expression in relation to overall cancer survival (left panel) and progression-free survival (right panel).

Supplementary Table 1: Percentage of positive MAD2 expression and MAD2 overexpression in numerous carcinoma types

Author	Cancer site(s)	% positive	Degree of MAD2 expression	Mean staining intensity	Mean weighted score
Agosten	Breast			2.1 for ductal carcinomas, 1.6 for lobular carcinomas.	
Burum-Auensen	Colorectal	59% (median)			
Burum-Auensen	Colorectal (UC-related)	32% (mean)			
Burum-Auensen	Testicular	15% intratubular germ cell neoplasia, 27% seminomas, 58% embryonal carcinomas, 52% yolk sac tumors, 30% teratomas			
Choi	Bladder	51%			
Du	Breast	49%			
Fung	Testicular		.	Mean nuclear intensity was lower ($p < 0.0001$) while mean cytoplasmic MAD2 staining intensity was higher ($p < 0.0001$) in seminoma tissues than normal tissues	
Genga	Myelodysplastic syndrome	87.5%	Higher MAD2 in MDS v. controls (28.3 ± 21.7% v. 5.96 ± 2.7%, respec-tively, $p < 0.001$)		
Gladhaug	Pancreatic	69.5% pancreatobiliary 77.9% Intestinal			
Hannisdal	Tonsillar	27% (median)			
Hisaoka	Sarcoma	52% TA, 66% non-TA			
Kato	Lung (2012)		Low/high expression in 80.7%/19.3% of adenocarcinoma Low/high expres-sion in 55.6%/44.4% of squamous cell carcinoma		
Kato	Lung (2011)	42.7%	Low/high expression in 73.7%/26.3% of squamous cell carcinoma		
Kim	Uterine cervical		Overexpression in 52.4% squamous cell carcinomas		
Ko	Salivary duct		High expression in 55.6%		
Li	Endometrial	85.71%			
Li	Colorectal (2003)	86.8%	Cancer/ normal ratio of MAD2 expres-sion = 2.07±1.87		
Li	Colorectal (2004)	75%			
Liao	Cervical	CIN = 64.62%,CINI =100%, CI-NII = 60.87%,CINIII = 48.15%, CC = 32%,SCC = 22.67%, AC = 40%	Staining intensity-barely detectable, easily observed, strong: CIN = 26.2/32.3/6.2%, CINI = 46.7/40/13.3%, CINII = 8.7/43.5/8.7%, CI-NIII = 29.6/18.5/0%, CC = 28/49/0%, SCC = 20/6.7/0%,AC = 40/0/0%		
McGrogan	Ovarian	93% (total), 34% (cytoplasmic)	Nuclear expression: 13% weak, 44% moderate, 36% strong		
Morishta	Uterine cervical		NAC+OP+R group: low/medium/high expression in 33.3/48.4/18.2% of cases. NAC+R group: low/medium/high ex-pression in 10/30/60% of cases		NAC+OP+R group: 4.5, NAC+R group: 8.2, CR+PR group: 4.9, SD +PD group:4.3
Nakano	Ovarian serous				Relapsed: 4.3, Re-lapse-free group:7.2
Nakano	Ovarian mucinous				Borderline malignant: 4.3, Malignant:7.6
Park	Ovarian		Low /high expression in 47.1/52.9% of cases		
Rizzardi	Oral SCC	100%	Overexpressed in 36.7% of cases		
Sotillo	Various		55.3% diffuse large B-cell lymphoma presented strong or moderate levels. MAD2 upregulation also seen in a subset of grade 3 follicular lymphomas, Burkitt's lymphomas and T- cell lym-phoblastic lymphomas		
Suraokar	Mesothelioma		Higher expression in cytoplasm of epitheloid tumours compared to the two other malignant pleural mesothelioma histotypes		
Teixeira	Oral cancer	100%	Mean labelling index of 64.90±18.70. in 61.1% tumour lesions. 42.6/33.3/24.1% of cases had stong/moderate/weak stain-ing. moderate and 24.1% weak staining		
Thoma	Renal and various		pVHL-negative ccRCC samples showed low MAD2 expression whereas high MAD2 expression was detected in ccRCC samples with strong pVHL expression		
Uemra	Esophageal SCC		Overexpression in 86% of cases		
Wang	Gastric	71%	Of MAD2 positive samples, 79% showed expression in the nucleus		
Yu	Osteosarcoma	100%	4.2% had cytoplasmic staining		
Zhao	Endometrial	85.7%			

Supplementary Table 2: Sensitivity analysis excluding individual studies from meta-analysis for highest v. lowest category of MAD2 protein levels and cancer progression

	Pooled risk estimate (95% CI)	I-squared (%)	*P*-value
Overall survival studies			
Excluding Burum Auensen *et al* 2008	1.402 (0.992–1.982)	73.0%	0.056
Excluding Choi *et al* 2013	1.271 (0.905–1.785)	72.0%	0.166
Excluding Furlong *et al* 2012	1.454 (1.053–2.008)	67.7%	0.023
Excluding Genga *et al* 2015	1.263 (0.921–1.730)	70.1%	0.147
Excluding Gladhaug (P) *et al* 2010	1.391 (0.967–2.002)	73.2%	0.075
Excluding Gladhaug (I) *et al* 2010	1.369 (0.966–1.941)	73.9%	0.078
Excluding Kato *et al* 2011	1.314 (0.914–1.889)	73.0%	0.140
Excluding Kim *et al* 2014	1.387 (0.985–1.952)	73.6%	0.061
Excluding Ko *et al* 2010	1.392 (0.990–1.957)	73.4%	0.057
Excluding Li *et al* 2013	1.239 (0.913–1.681)	67.7%	0.170
Excluding Nakano (S) *et al* 2012	1.444 (1.055–1.976)	68.9%	0.022
Excluding Nakano (M) *et al* 2012	1.356 (0.967–1.903)	74.0%	0.078
Excluding Suraokar *et al* 2014	1.331 (0.924–1.916)	73.7%	0.124
Excluding Yu *et al* 2010	1.263 (0.910–1.752)	71.5%	0.163
Excluding Zhang *et al* 2008	1.365 (0.913–2.043)	73.8%	0.130
Progression- free survival studies			
Excluding Furlong *et al* 2012	0.715 (0.374–1.365)	69.8%	0.309
Excluding Kato *et al* 2011	0.506 (0.355–0.721)	0%	0.000
Excluding Kim *et al* 2014	0.623 (0.314–1.237)	78.6%	0.176
Excluding Ko *et al* 2010	0.624 (0.317–1.226)	78.6%	0.171
Excluding McGrogan *et al* 2014	0.669 (0.336–1.335)	77.1%	0.254
Excluding Nakano *et al* 2012	0.703 (0.367–1.346)	76.1%	0.287
Excluding Park *et al* 2013	0.719 (0.388–1.332)	75.8%	0.294
